# RETRACTION: Effectiveness of Nursing Intervention in the Operating Room to Prevent Wound Infections in Patients Undergoing Orthopaedic Surgery: A Meta‐Analysis

**DOI:** 10.1111/iwj.70310

**Published:** 2025-03-06

## Abstract

**RETRACTION:** ZhuD. and LuoQ. , “Effectiveness of Nursing Intervention in the Operating Room to Prevent Wound Infections in Patients Undergoing Orthopaedic Surgery: A Meta‐Analysis,” International Wound Journal 20, no. 11 (2023): 4103–4111, 10.1111/iwj.14304.37433641 PMC10681532

The above article, published online on 11 July 2023, in Wiley Online Library (http://onlinelibrary.wiley.com/), has been retracted by agreement between the journal Editor in Chief, Professor Keith Harding; and John Wiley & Sons Ltd. Following an investigation by the publisher, all parties have concluded that this article was accepted solely on the basis of a compromised peer review process. The editors have therefore decided to retract the article. The authors did not respond to our notice regarding the retraction.

**RETRACTION:** D. Zhu and Q. Luo , “Effectiveness of Nursing Intervention in the Operating Room to Prevent Wound Infections in Patients Undergoing Orthopaedic Surgery: A Meta‐Analysis,” International Wound Journal 20, no. 11 (2023): 4103–4111, 10.1111/iwj.14304.37433641 PMC10681532

The above article, published online on 11 July 2023, in Wiley Online Library (http://onlinelibrary.wiley.com/), has been retracted by agreement between the journal Editor in Chief, Professor Keith Harding; and John Wiley & Sons Ltd. Following an investigation by the publisher, all parties have concluded that this article was accepted solely on the basis of a compromised peer review process. The editors have therefore decided to retract the article. The authors did not respond to our notice regarding the retraction.

